# Resonance-Dependent Pattern Dynamics in a Neural Field for Spatial Coding

**DOI:** 10.3390/biomimetics11040224

**Published:** 2026-03-24

**Authors:** Yani Chen, Youhua Qian, Jigen Peng

**Affiliations:** 1 Machine Life and Intelligence Research Center, Guangzhou University, Guangzhou 510006, China; 1112215003@e.gzhu.edu.cn; 2 School of Mathematics and Information Science, Guangzhou University, Guangzhou 510006, China; 3 School of Mathematical Sciences, Zhejiang Normal University, Jinhua 321004, China

**Keywords:** navigation system, spatial representation, pattern dynamics, population manifold

## Abstract

Continuous representations in brain navigation system are manifested as spatially structured patterns of population activity, such as a single-peaked bump moving along a ring manifold in head-direction system and hexagonal lattice patterns underlying spatial representation in grid-cell systems. These phenomena are commonly modelled within the framework of continuous attractor networks (neural dynamical field), yet the mechanisms by which activation-function nonlinearities interact with connectivity structure to determine pattern selection and dynamics remain incompletely understood. This paper separately analyses the interactions between non-resonant and resonant modes using a multiscale unfolding approach. We show that, when the critical modes satisfy a resonance condition, the quadratic nonlinearity of the activation function induces a three-mode coupling that fundamentally alters the structure of the amplitude equations and becomes the dominant mechanism governing spatial pattern selection. Building on this analysis, we introduce a weak asymmetric component in the connectivity and analytically derive the resulting pattern drift velocity, which is subsequently confirmed by numerical simulations. Finally, we apply these dynamical mechanisms to input-driven scenarios, illustrating that similar dynamical mechanisms can account for activity-bump tracking in head-direction models and lattice translations in grid-cell models. Overall, this work provides an analytically tractable framework for studying pattern dynamics in neural field models relevant to spatial representations, and may inform biomimetic approaches to spatial representation and navigation.

## 1. Introduction

Numerous key representations in the brain navigation system are manifested as spatially structured patterns of population activity, such as a single-peaked ‘bump’ on a ring and hexagonal lattice patterns. These spatial structures are closely related to spatial cognition and navigation [1,2,3]. In particular, population activity in the head-direction system [4,5] can be effectively described as the continuous movement of a single-peaked bump along a ring attractor manifold [6,7,8,9,10], whereas population activity in the grid-cell system is often regarded as the continuous evolution of an attractor state over a two-dimensional representational manifold [3,11,12]. A large body of work on continuous attractor network model have successfully captured key features of path integration and spatial localisation [1,7,11,13]. Together, these studies indicate that the stable geometric structure of these spatial patterns, and their continuous movement within an internal representational space under behaviourally relevant inputs, constitutes a key dynamical principle supporting heading tracking and position updating during navigation [1,9,10].

In continuous attractor model (neural field model), such spatial structures emerge from relatively simple dynamical mechanism. One common scenario is that, as a system parameter is varied, a spatially uniform activity state first undergoes a Turing instability, giving rise to spatially periodic population patterns [14,15,16,17]. Early studies have investigated such mechanism in one-dimensional neural field models, including the existence and stability of travelling solutions [18,19,20,21,22,23]. In two-dimensional situation, Turing instabilities can generate richer spatial patterns, such as hexagonal lattices, structure and bifurcation properties have been characterized in numerous theoretical studies [15,16,17,24]. In addition, a number of works have introduced adaptation, or additional slow variables to explore travelling or drifting structures [22,25,26,27,28]. Together, these studies reveal the rich spatiotemporal dynamics supported by neural field models.

However, in a basic framework of continuous attractor and neural field models, it remains unclear which key mechanisms dominate spatial pattern, and how these mechanisms may relate to spatial representations observed in biological navigation systems. Motivated by this question, we investigate a two-dimensional neural field model that relies solely on the connectivity kernel and the intrinsic activation nonlinearity, without introducing additional dynamical variables. In this setting, we examine how resonant interactions between critical modes give rise to triad coupling induced by quadratic nonlinearities, thereby altering the structure of the amplitude equations and ultimately influencing the selection of stable spatial patterns [15,16,17]. We further analyse, in the context of two-dimensional periodic patterns, how weak geometric asymmetries in the connectivity structure are translated into predictable pattern drift velocities. Finally, we explore how these dynamical mechanisms can be related, within a common dynamical framework of attractor-based representations, to activity tracking in head-direction systems and to lattice-like representations in grid-cell networks [5,7,9,11,12].

To address these questions, following classical multiscale approaches developed in the neural field and pattern formation literature, we derive amplitude equations near criticality and separately consider non-resonant and resonant regimes of modal interaction [15,16,17,24]. By comparing the structures of these two classes of amplitude equations, we identify a key coupling coefficient, jointly determined by the quadratic nonlinearity of the activation function and the spectral properties of the connectivity kernel, which quantitatively characterises the strength of interactions between modes. Numerical simulations further confirm the central role of this coefficient in determining the emergence of stripe versus hexagonal patterns. Building on these results, we introduce weak geometric asymmetries in the connectivity kernel and derive analytical expressions for the resulting pattern drift velocities within the weakly nonlinear framework, demonstrating that, at the critical wavenumber, these velocities are primarily controlled by the asymmetry parameter itself and are largely insensitive to the detailed shape of the kernel. Numerical results validate the robustness of this analytical prediction across different pattern types. Finally, we apply the theoretical mechanisms to behaviourally driven scenarios in head-direction and grid-cell systems, showing that the model reproduces population-level activity representations consistent with experimental observations, thereby providing a dynamical framework for interpreting spatial activity patterns observed in neural navigation systems [4,5,6,11,12].

## 2. Model Description

In this study, we consider a continuous stimulus encoded by an ensemble of neurons. The stimulus may, for example, represent movement direction or spatial position. We model only the population-averaged activity, which can be written as the scalar neural field equation [17]:

(1) $$\tau \frac{\partial u(x,t)}{\partial t}=-u\left(x,t\right)+\mu \int_{R^{2}}\omega_{a}\left(x,x^{\prime }\right)F\left(u\left(x^{\prime },t\right)\right)dx^{\prime },$$

with τ=1. Here x denotes the preferred stimulus (or feature) coordinate of a neuron in the continuous neural field, and u(x,t) represents the population-averaged synaptic input or activity level at location x and time *t*. A global gain parameter μ is treated as a bifurcation parameter in the neural field equation, and is expanded about its critical value $\mu_{c}$ in the analysis below.

(2) $$F\left(u\right)=\frac{1}{1+e^{-b(u+\delta )}}-\frac{1}{1+e^{-b\delta }}.$$

The activation function *F* is modeled as a smooth sigmoid function, a standard assumption in rate-based neural field models that captures the thresholding and saturation properties of neuronal input–output relationships. The theoretical results derived in this work primarily depend on the local derivatives of *F* at the operating point (e.g., $F^{\prime }\left(u_{0}\right),F^{\prime \prime }\left(u_{0}\right),F^{\prime \prime \prime }\left(u_{0}\right)$). Therefore, qualitatively similar results would be expected for other smooth activation functions with comparable nonlinear characteristics. The parameters *b* and δ respectively control the neuronal gain and the effective activation threshold.

The convolution kernel is used to describe lateral interactions, in the neural field and is commonly chosen as a combination of short-range excitation and longer-range inhibition, a structure known as Mexican-hat connectivity. The parameter a in our model can be interpreted as a velocity- or rotation-driven input in real time. Such a convolution kernel can destablise the system to generate spatial pattern formation, analogous to a Turing instability in reaction-diffusion systems. As a representative example, we consider a difference-of-Gaussians kernel of the form

(3) $$\begin{array}{c} \omega \left(|x|\right)=e^{-\frac{x^{2}}{2}}-Ae^{-\frac{x^{2}}{2\sigma^{2}}}, \end{array}$$

where A>0 and σ>1 control the relative strength and spatial extent of inhibition. The shifted kernel is defined as $\omega_{a}\left(x\right)=\omega \left(x-a\right)$, or equivalently $\omega_{a}\left(x-x^{\prime }\right)=\omega \left(\left(x-x^{\prime }\right)-a\right)$. This kernel is asymmetric when a≠0 and reduces to a symmetric kernel when a=0.

**Figure 1:**
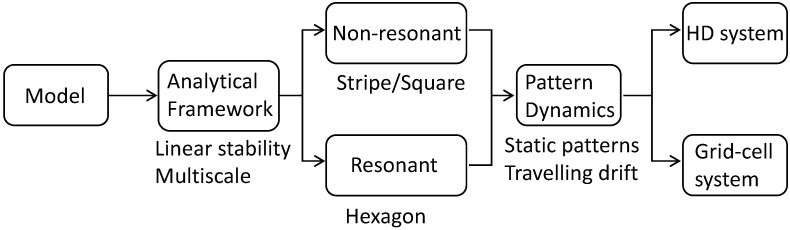
Overview of the analytical framework and simulation in this study. The analysis distinguishes (i) resonant versus non-resonant mode interactions, which determine pattern selection and stability (e.g., stripe/square versus hexagonal states), and (ii) symmetric versus weakly asymmetric connectivity, controlled by the parameter **a**, which governs whether the selected patterns remain stationary or undergo drift. The theoretical predictions are subsequently validated through numerical simulations in navigation-related neural systems.

To provide an overview of the analytical framework and simulations in this study, Figure 1 summarizes the main methodological steps, including model formulation, linear stability analysis, weakly nonlinear expansion, and the resulting pattern dynamics. Resonant and non-resonant interactions determine pattern selection, while the asymmetry parameter **a** induces a drift that transforms stationary patterns into travelling ones. Numerical simulations are used to verify the theoretical predictions.

## 3. Results

In this section, we first characterise the modal lateral interactions that govern the selection of stripe versus hexagonal patterns near the bifurcation point, using a weakly nonlinear framework to obtain reduced amplitude equations. We then identify the key contribution to pattern drift velocity and validate its consistent to the realistic inputs through a combination of analytical arguments and numerical simulations. Finally, to connect these pattern-forming mechanisms with biologically relevant scenarios, we present two simulations demonstrating how the model reproduces heading-direction signals and grid-cell lattice responses.

### 3.1. Multiple Scale Analysis

To analyse the dynamics near bifurcation, we apply the method of multiple scales. We consider a spatially homogeneous fixed point and focus on the symmetric case a=0. For the sigmoidal activation function in Equation (2), one has F(0)=0. Hence Equation (1) admits the trivial homogeneous fixed point $u_{0}=0$, which we take as the reference state for the subsequent analysis (the additional non-trivial homogeneous solutions is not considered here). Although additional non-trivial homogeneous solutions may exist for sufficiently large gain, the present analysis focuses on the loss of stability of the trivial resting state and the onset of spatial pattern formation. This choice allows us to isolate the primary Turing-type instability and the associated modal interactions.

More generally, a spatially fixed point $u_{0}$ of Equation (1) must satisfy the self-consistency condition

(4) $$u_{0}={\hat{\omega }}_{0}F\left(u_{0}\right),\;\;{\hat{\omega }}_{0}=\int_{R^{2}}\omega \left(x\right)dx.$$

Expanding the activation function about $u_{0}=0$ gives

(5) $$F\left(u\right)=F\left(u_{0}\right)+F^{\prime }\left(u_{0}\right)u+\frac{1}{2}F^{\prime \prime }\left(u_{0}\right)u^{2}+\frac{1}{6}F^{\prime \prime \prime }\left(u_{0}\right)u^{3}+O\left(u^{4}\right),$$

where we denote $s=F^{\prime }\left(u_{0}\right)$, $f_{2}=F^{\prime \prime }\left(u_{0}\right)$ and $f_{3}=F^{\prime \prime \prime }\left(u_{0}\right)$.

Linearising Equation (1) about fixed point $u_{0}$ and writing $u\left(x,t\right)=u_{0}+\rho \left(x,t\right)$, yields

(6) $$\partial_{t}\rho =L_{\mu }\rho +O\left(\rho^{2}\right),\;L_{\mu }=-I+\mu s\;W*,$$

where $\left(W*u\right)\left(x\right)=\int \omega \left(\right|x-x^{\prime }\left|\right)u\left(x^{\prime }\right)dx^{\prime }$. Seeking modal solutions $\rho \left(x,t\right)=e^{\lambda t}p\left(x\right)$ yields the eigenvalue problem

(7) $$\lambda p\left(x\right)=-p\left(x\right)+\mu s\int_{R^{2}}\omega (|x-x^{\prime }\left|\right)p\left(x^{\prime }\right)\;dx^{\prime }.$$

Since the kernel is translation-invariant (convolution), the eigenfunctions of linear operator $L_{\mu }$ are Fourier modes $p\left(x\right)=e^{ik\cdot x}$. For an isotropic kernel ω(x)=ω(|x|), the corresponding eigenvalues depend only on the wavenumber k=|k|, leading to the dispersion relation

(8) $$\lambda \left(k\right)=-1+\mu s\hat{\omega }\left(k\right),$$

where $\hat{\omega }\left(k\right)=\int \omega \left(x\right)e^{-ik\cdot x}\;dx$ denotes the Fourier transform of the kernel evaluated at k=|k|. It is straightforward to determine conditions of $u_{0}$ losing stability, leading to the formation of spatially periodic patterns. The Fourier transform of Equation (3) is $\hat{\omega }\left(k\right)=2\pi e^{-\frac{k^{2}}{2}}-2\pi \sigma^{2}Ae^{-\frac{1}{2}\sigma^{2}k^{2}}$. As μ increases to $\mu =\mu_{c}=\frac{1}{s\hat{\omega }\left(k_{c}\right)}$, the dispersion curve λ(k) passes through zero at $k=k_{c}$, where $\hat{\omega }\left(k_{c}\right)=\max_{k}\hat{\omega }\left(k\right)$.

#### 3.1.1. Derivation of Amplitude Equations for Stationary Solutions

To capture the slow evolution of the weak non-linear amplitude near the bifurcation point, a small parameter ε is introduced and

(9) $$\begin{array}{c} \mu =\mu_{c}+\varepsilon^{2}\mu_{2},\\ \partial_{t}\mapsto \partial_{t}+\varepsilon^{2}\partial_{T},\\ \rho \left(x,t\right)=\varepsilon u_{1}+\varepsilon^{2}u_{2}+\varepsilon^{3}u_{3}+\ldots , \end{array}$$

where we introduce a slow time $T=\varepsilon^{2}t$, and treat *t* and *T* as independent variables. In the non-resonant case, quadratic interactions generate only non-critical modes, so that the $O(\varepsilon^{2})$ correction can be uniquely slaved to $u_{1}$, and the amplitude dynamics arise at $O(\varepsilon^{3})$.

Collecting ε-items by non-linear expansion, we obtain

(10) $$\begin{array}{c} O\left(\varepsilon \right):\;\partial_{t}u_{1}=-u_{1}+\mu_{c}sW*u_{1}, \end{array}$$

where $\left(W*u\right)\left(x\right)=\int \omega \left(x-x^{\prime }\right)u\left(x^{\prime }\right)dx^{\prime }$, and W∗ denotes convolution with kernel ω. For critical modes satisfying $\lambda (k_{c})=0$, the leading-order dynamics is neutrally stable on the fast time scale, so that $\partial_{t}u_{1}=0$. Consequently $u_{1}$ lies in the null space of the linear convolution operator $I-\mu_{c}sW*$, that is $u_{1}$ spanned by waves with modulus $k_{c}$,

(11) $$\begin{array}{c} u_{1}\left(x,T\right)=\sum_{n=1}^{N}\left(C_{n}\left(T\right)e^{ik_{n}\cdot x}+C_{n}^{*}\left(T\right)e^{-ik_{n}\cdot x}\right),\;\left|k_{n}\right|=k_{c}. \end{array}$$

For non-resonant interactions, involving two competing critical modes $C_{1}$ and $C_{2}$, the amplitude equations take the generic form

(12) $$\begin{array}{c} \partial_{T}C_{1}=C_{1}\left(\gamma -\alpha {\left|C_{1}\right|}^{2}-2\beta {\left|C_{2}\right|}^{2}\right),\\ \partial_{T}C_{2}=C_{2}\left(\gamma -\alpha {\left|C_{2}\right|}^{2}-2\beta {\left|C_{1}\right|}^{2}\right), \end{array}$$

where the coefficients α,β depend on the activation nonlinearity and the kernel spectrum (see Appendix A). For the stripe solution, the equal amplitude *R* satisfies $R(\gamma -\alpha |R{|}^{2})=0$. A stable non-trivial branch emerges with $\left|R\right|=\sqrt{\frac{\gamma }{\alpha }}$. For the square solution, the equal amplitude *R* satisfies $R(\gamma -\left(\alpha +2\beta \right)|R{|}^{2})=0$, leading to a non-trivial solution $\left|R\right|=\sqrt{\frac{\gamma }{\alpha +2\beta }}$.

For hexagonal patterns, three critical wavevectors $\left\{k_{1},k_{2},k_{3}\right\}$ satisfy $k_{1}+k_{2}+k_{3}=0$. In this case, quadratic nonlinearities generate resonant interactions that project directly onto the critical subspace. To capture this effect, we introduce a faster slow time $T_{1}=\varepsilon t$, $\partial_{t}\mapsto \partial_{t}+\varepsilon \partial_{T_{1}}+\varepsilon^{2}\partial_{T_{2}}$, and $\mu =\mu_{c}+\varepsilon \mu_{1}+\varepsilon^{2}\mu_{2}$. At $O(\varepsilon^{2})$, the quadratic term arising from the Taylor expansion of the activation function produces resonant contributions proportional to $C_{n-1}^{*}C_{n+1}^{*}$ at the critical wavevectors. A detailed derivation of the associated coefficients is provided in Appendix B.

Collecting the resulting solvability conditions yields the amplitude equations for hexagonal patterns in the unified form

(13) $$\partial_{T_{1}}C_{n}=C_{n}\left(\gamma -\alpha |C_{n}{|}^{2}-2\beta (|C_{n-1}{|}^{2}+{\left|C_{n+1}\right|}^{2})\right)-\eta \;C_{n-1}^{*}C_{n+1}^{*},$$

where n=1,2,3(mod3) and $\eta =\mu_{c}f_{2}\hat{\omega }\left(k_{c}\right)$.

For the hexagonal solution, the equal amplitude *R* satisfies $R(\gamma -\left(\alpha +4\beta \right)|R{|}^{2}-\eta R)=0$. Consider a non-trivial branch with $\left|R\right|=\frac{-\eta \pm \sqrt{\eta^{2}+4\left(\alpha +4\beta \right)\gamma }}{2(\alpha +4\beta )}$. To sum up, η quantifies how the curvature of the activation function (through $F^{\prime \prime }\left(u\right)$) interacts with the synaptic kernel $W_{k_{c}}$ to mediate triad interactions. Small |η| implies weak modal coupling, favouring single-mode stripe patterns, whereas large |η| facilitates phase-locked hexagonal configurations.

#### 3.1.2. Derivation of TravelLing Solution Velocity

We now introduce a small spatial shift a breaks symmetry convolution kernel, that is $\omega_{a}\left(x\right)=\omega \left(x-a\right),\;a\neq 0$. This is equivalent to multiplication by a phase factor in the Fourier domain,

(14) $$\begin{array}{c} {\hat{\omega }}_{a}\left(k\right)=e^{-ik\cdot a}\hat{\omega }\left(k\right), \end{array}$$

and the linearized spectrum

(15) $$\begin{array}{c} \lambda \left(k\right)=-1+\mu se^{-ik\cdot a}\hat{\omega }\left(k\right), \end{array}$$

where $e^{-ik\cdot a}=1-i\left(k\cdot a\right)-\frac{1}{2}{\left(k\cdot a\right)}^{2}+\ldots$. Then we obtain

(16) $$\begin{array}{c} \lambda \left(k\right)=-1+\mu s\hat{\omega }\left(k\right)-i\mu s\left(k\cdot a\right)\hat{\omega }\left(k\right)+{O(|a|}^{2}). \end{array}$$

The asymmetric phase factor introduces a non-zero imaginary component to the spectrum, implying a drift of the spatial mode. Separating real and imaginary parts, we obtain

(17) Reλ(k)=−1+μsω^(k),Imλ(k)=−μs(k·a)ω^(k),

with k=|k|. The resulting drift (group) velocity of the mode is therefore

(18) vg=∇kImλ(k)=−μsω^(k)a+(k·a)ω^′(k)kk.

Evaluating on the critical circle $\left|k\right|=k_{c}$, where ${\hat{\omega }}^{\prime }\left(k_{c}\right)=0$, gives

(19) $$\begin{array}{c} v_{g}\approx -\mu sa\hat{\omega }\left(k_{c}\right). \end{array}$$

At the instability threshold, $-1+\mu_{c}s\hat{\omega }\left(k_{c}\right)=0$, hence $v_{g}=-a$ at onset.

This shows that introducing a small spatial displacement a in the kernel induces a travelling whose drift velocity is set by −a at criticality, independent of the specific shape of kernel.

### 3.2. Simulation of Model’s Dynamical Behaviour

To validate our theoretical solution, we conduct some numerical simulation within model, basic parameters are selected as Table 1.

#### 3.2.1. Static Solution

**Figure 2:**
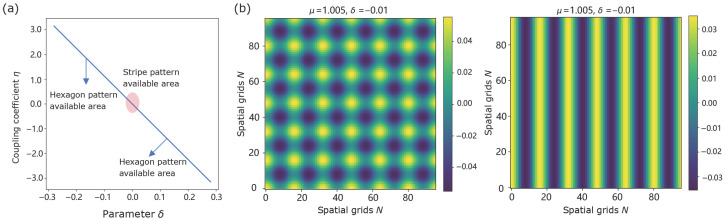
Transition from a static square solution to a stripe solution with μ=1.005. (**a**) The system tend to form stripe patterns when the coupling coefficient is small, whereas larger coupling promotes the emergence of hexagonal patterns. (**b**) With δ=−0.01, the system evolves from a square pattern into a stripe pattern, indicating the intrinsic instability of the square configuration.

**Figure 3:**
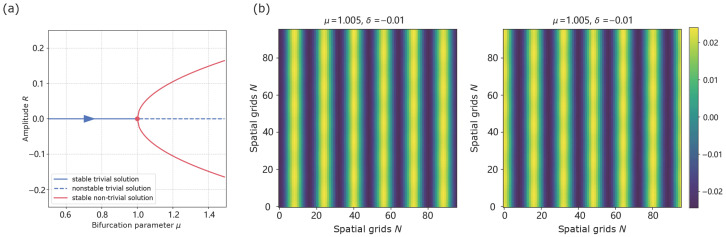
Static stripe solution with μ=1.005,δ=−0.01. (**a**) Near onset, the amplitude equation admits a supercritical pitchfork with $\pm \sqrt{\frac{\gamma }{\alpha }}$, illustrating the stripe amplitude in the weakly nonlinear regime. (**b**) Phase diagrams under opposite initial conditions reveal complementary regions of high response (yellow part). The simulated amplitudes are in close agreement with the theoretical prediction $\sqrt{\frac{\gamma }{\alpha }}$.

**Figure 4:**
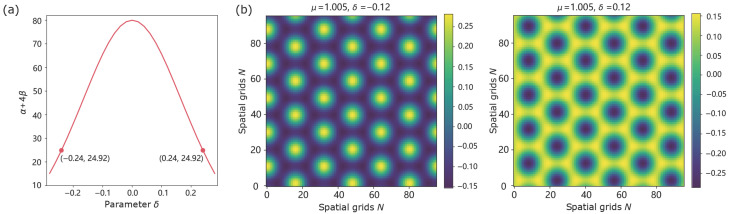
Static hexagonal solution with μ=1.005. (**a**) The weakly nonlinear amplitude equation admits a branch of hexagonal solutions whose existence and structure are governed by the balance between the quadratic triad interaction η and the cubic saturation α+4β. Here, we select δ=0.12 (as motivated by Figure 2a), which facilitates the formation of hexagonal patterns. (**b**) Phase diagrams with δ=±0.12 and identical initial conditions demonstrate a phase reverse: regions of high response (highlighted circles) appear on the left in one case but on the right in the other, reflecting the effect of sign reversal in η.

A crucial parameter η, manifests as a resonance phenomenon in the system, indicating a bias in pattern selection for stripe patterns at small |δ| and for hexagonal patterns at larger |δ| (Figure 2a). To test the existence of static stripe solution, we select μ=1.005,δ=−0.01, and we observe the emergence of two symmetry-related stripe states with opposite phases, consistent with the pitchfork structure predicted by the amplitude equation. Figure 3 illustrates two symmetry-related stripe states, where opposite initial conditions lead to stripe patterns with opposite phases. When μ=1.005,δ=±0.12, the system demonstrates a phase reverse in Figure 4, which shows regions of high response (highlighted circles) shaping circle on the left and supplementary on the right, consistent with the expected effect of the sign of η. We observe that square-like patterns are typically transient in our simulations and eventually evolve into either stripe or hexagonal configurations.

#### 3.2.2. Travelling Solution

**Figure 5:**
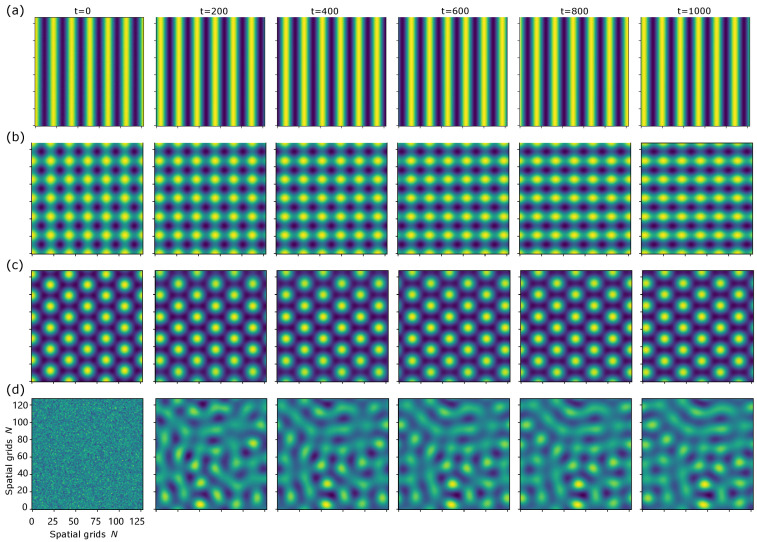
Travelling phase diagrams with a=(0.12,0) at t=0,200,400,600,800,1000. Parameters settings: (**a**,**b**) μ=1.01,δ=−0.01. (**c**,**d**) μ=1.04,δ=−0.24. To focus on the pattern travelling phenomena, initial conditions has been elaborate designed to yield stripe-, square- or hexagonal-type states. As in Figure 2, the square pattern in (**b**) appears to shrink towards a stripe solution over time.

The introduction of asymmetric components a induces travelling dynamics in the patterns. Figure 5 illustrates travelling behaviour in stripe, square, hexagonal, and irregular patterns, each initialised from stripe, square, hexagonal or random states to accelerate convergence towards stable solutions. Consistent with the above analysis, the square pattern eventually evolves into the stripe pattern, although such tendency is only partially visible in Figure 5b.

**Figure 6:**
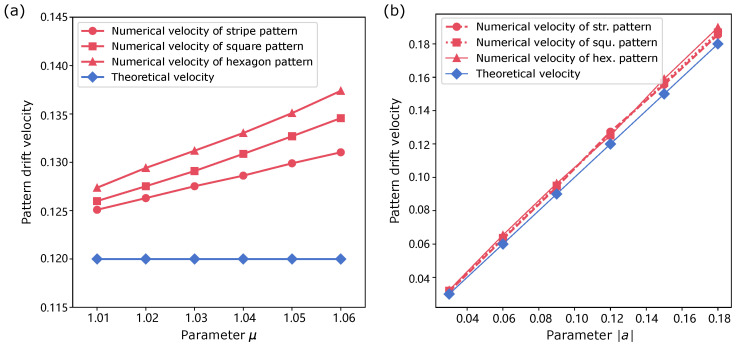
Relationship between pattern drift velocity and parameters μ,δ. (**a**) Drift velocity as a function of the gain parameter μ for different spatial patterns. Across all cases, increasing μ leads to a systematic increase in the travelling speed. The stripe solution exhibits the closest agreement with theory, while deviations observed for hexagonal patterns reflect higher-order effect. (**b**) As |a| increases, the numerical drift velocities remain consistent with theoretical values across all patterns.

To examine the disparity between the numerical drift velocity and the theoretical velocity of patterns as μ departs from the critical point, we present the results in Figure 6a. We find that the numerical travelling velocities diverge from theoretical prediction as μ increases. Among the three types, the stripe solution is the most proximity to the theoretical value, reflecting the velocity accuracy effected by the number of modes. In Figure 6b, as |a| increases, the numerical drift velocities of all patterns remain consistent with theoretical values.

#### 3.2.3. Illustrative Examples of Attractor Dynamics in Navigation-Related Circuits

To illustrate how the dynamical mechanisms identified in this work may relate to navigation-related neural systems, we present two simulations, such as moving bumps in the heading-direction system [7] and travelling hexagonal lattices [11] in the grid-cell system. To evaluate the performance of our model in a physically realistic setting, we implemented two simulations: one for a population of heading-direction cells and another for a population of grid cells.

For the heading-direction cell population, which requires a one-dimensional continuous attractor, we assume $\tau \frac{\partial u(x,t)}{\partial t}=-u\left(x,t\right)+\int_{R}\omega_{a}\left(x,x^{\prime }\right)F\left(u\left(x^{\prime },t\right)\right)dx^{\prime }$ with N=50 and $m_{\lambda }=1$, where u(x,t) denotes the activity of neurons tuned to preferred orientation. Neurons #1–50 uniformly represent the angular range (−π,π). We set the initial orientation at 0 (corresponding to neuron #25). In biological systems, head rotation is encoded by vestibular inputs providing an angular velocity signal. In continuous attractor models, such velocity signals are commonly implemented by introducing a small asymmetry in the recurrent connectivity, which drives a translational motion of the activity bump along the attractor manifold.

**Figure 7:**
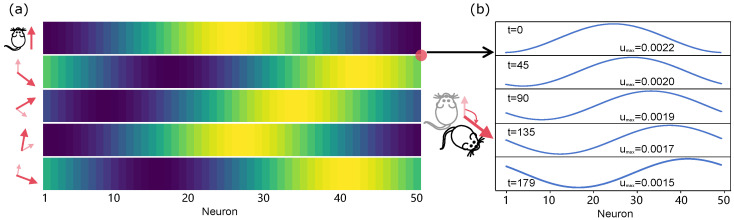
The bump tracks head orientation in heading-direction system. (**a**) Right column: schematic of head rotation. Left column: initial heading direction followed by four rotations, indicated from small semitransparent arrows to large opaque arrows. Model inputs represent normalised rotation signals derived from sensory organs (e.g., vestibular). Middle column: x-axis represent numbers of neurons (#1–50), corresponding to (−π,π). The activity bump (yellow region) tracks the head rotation in both clockwise and anticlockwise directions. (**b**) In each bump transition, the one-dimensional pattern driven by the asymmetric components, travelling smoothly to the corresponding orientation. The time *t* denotes simulation steps, instead of real physical time.

In our model, this effect is captured by the parameter a, which controls the degree of asymmetry in the connectivity kernel and is proportional to the instantaneous angular velocity of head rotation. To illustrate the robustness of heading tracking, we adopt a piecewise-constant angular velocity input, where a is held constant over successive time intervals. Specifically, we use the sequence a=[0.2,−0.1,−0.08,0.16,−0.1], where each value represents a normalized angular velocity during a fixed time window, and the sign of a indicates the direction of rotation. This sequence corresponds to a head-rotation trajectory involving multiple changes in rotational direction. As shown in Figure 7b, under these inputs the activity bump travels smoothly along the ring attractor, accurately tracking the corresponding head orientation. Importantly, the bump dynamics remain stable and continuous across changes in the sign of a, demonstrating that the model robustly encodes heading direction over time.

**Figure 8:**
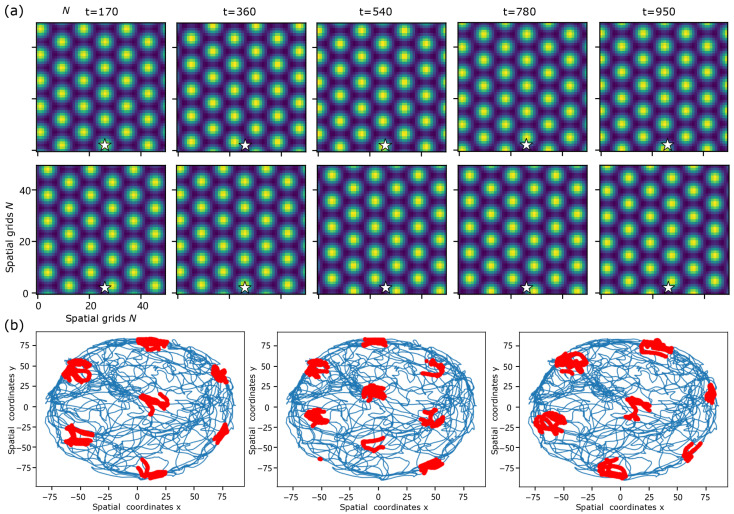
Travelling patterns and supra-threshold activity event maps in a grid-cell model. The trajectory of a rat running in a square arena (170 cm × 170 cm) is used to generate velocity inputs [29]. Here *t* denotes physical time. The model receives velocity inputs in real time, which introduce asymmetric components in the recurrent connectivity and lead to travelling activity patterns. (**a**) Snapshots of the population activity at t=170,360,540,780,950, with the star marking neuron #126. Upper panels show unscaled velocity inputs; lower panels show inputs scaled by a factor of 1.3. (**b**) Spatial distribution of supra-threshold activity events for neuron #126. Blue curves indicate the rat trajectory. Red dots mark spatial locations at which the activity of neuron #126 exceeds 60% of its maximum value along the trajectory. Left: unscaled velocity inputs. Right: velocity inputs scaled by a factor of 1.3 and rotated by $45^{\circ }$, resulting in a correspondingly rotated hexagonal event map.

**Figure 9:**
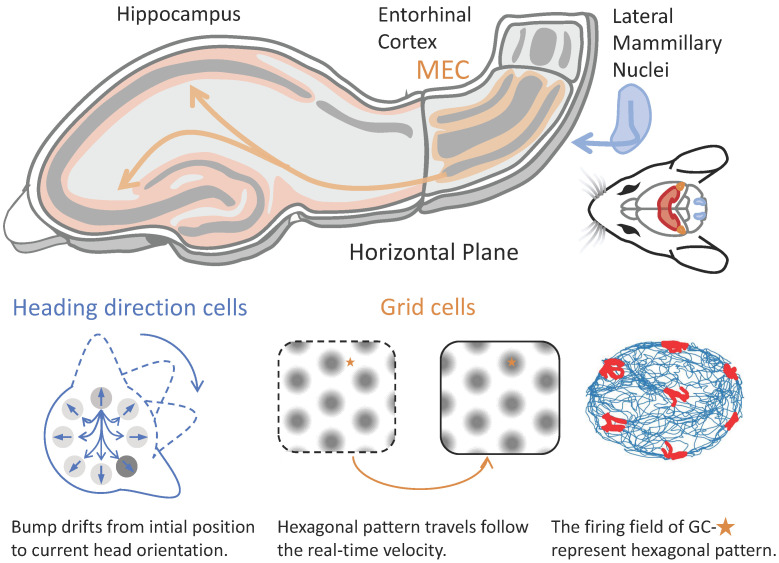
Conceptual sketch linking travelling activity patterns to rodent navigation systems. Upper: A schematic illustration [30,31] of functional interactions among head-direction-related brain regions and downstream spatial representations. For simplicity, only major pathways are shown (e.g., lateral mammillary nuclei, LMN, to entorhinal cortex, EC, and hippocampus), without implying a complete or exclusive anatomical circuitry. The diagram is intended as a conceptual illustration rather than a detailed anatomical model. Lower: In head-direction-related circuits, population activity forms a travelling bump that continuously tracks the animal’s heading. In the entorhinal cortex, analogous population-level dynamics give rise to hexagonal lattice firing patterns in grid cells, which can be interpreted as translations of an underlying periodic activity pattern driven by velocity-related inputs.

For the grid-cell population, which requires a two-dimensional self-organizing continuous attractor, we assume velocity inputs $a=(a_{x},a_{y})$ derived from the rodent trajectories [29]. Specifically, we use the trajectory of a rat running in a square arena (170 cm × 170 cm). The model receives real-time velocity inputs, which introduce small asymmetric components in the recurrent connectivity and thereby induce travelling activity patterns (Figure 8a). To visualise the spatial structure of activity for an individual unit, we focus on neuron #126 and record its activity $u_{126}\left(t\right)$ along the animal trajectory. We identify supra-threshold activity events by selecting time points at which

(20) $$\begin{array}{c} u_{i}\left(t\right)>g\max_{t}u_{i}\left(t\right), \end{array}$$

with g=0.6. The corresponding spatial locations of the animal at these time points are marked in red in Figure 8b. These event locations form a hexagonal lattice, reflecting the underlying travelling hexagonal activity pattern of the neural field. The formation of event locations for #126 in Figure 8b can be interpreted as follows: when the rat is at t=170 at position (13.5,82.97) cm, the neuron fired; then when the rat at t=360 at position (45.98,54.92) cm, the neuron silent. Finally, the responses of neuron #126 form hexagonal lattice. By applying velocity inputs with different scaling factors, the model generates lattices of varying spatial scales. When the velocity input was rotated by 45°, the translational direction of the pattern also rotated by 45°, thereby activating a different set of lattice nodes and yielding a hexagonal firing map rotated by 45° (see Figure 8b (right)). To provide a conceptual illustration of how the modeled dynamics relate to navigation-related circuits, we illustrate the inner-regional connections, as shown in Figure 9, thereby highlighting the application of different solution types in various cortex.

## 4. Discussion

In this work, by combining bifurcation analysis with weakly nonlinear theory, we identified the conditions under which stationary, travelling, and hexagonal patterns emerge, and clarified how velocity-driven asymmetries and allocentric anchoring constrain their stability and phase structure. These results provide a unified dynamical framework for understanding both biological spatial representations and their biomimetic applications in artificial navigation systems.

### 4.1. Effective Connectivity Hypothesis in Navigation Circuits

Some experimental studies suggest that the anatomical connectivity in navigation circuits does not conform to a classical Mexican-hat structure. For example, neurons in the head-direction system appear to form highly local circuits rather than long-range excitatory connections [32], and recurrent interactions among grid cells are thought to be mediated predominantly through inhibitory interneurons rather than direct excitation [33]. From an anatomical perspective, therefore, the classical Mexican-hat connectivity pattern should not be interpreted as synaptic connections. However, in neural dynamical models the connectivity kernel is more appropriately interpreted as an effective connectivity. In networks containing inhibitory interneurons, multi-step pathways involving inhibition and disinhibition can give rise to effective interaction profiles that resemble local amplification combined with longer-range suppression at the population level. Consequently, even if the underlying circuitry is dominated by inhibitory connections, the emergent population dynamics may still exhibit coupling structures functionally similar to a Mexican-hat kernel.

This perspective has long been adopted in theoretical studies of continuous attractor networks. In the neural field framework introduced by Amari [14], local excitation together with long-range inhibition was shown to stabilise spatially localised activity patterns. Subsequently, Zhang [7] employed a similar connectivity structure to model the stable activity bump underlying head-direction coding. In models of grid-cell networks, Burak and Fiete [11] further demonstrated that such effective interactions can give rise to two-dimensional lattice patterns resembling grid-cell firing fields.

In the present study we adopt this classical form as an abstract description of the connectivity kernel. Importantly, our goal is not to reconstruct the detailed microcircuitry of the entorhinal cortex. Rather, we focus on how the shape of the connectivity kernel, nonlinear activation functions, and resonant interactions jointly determine pattern formation and dynamics in neural fields. From this perspective, the Mexican-hat kernel provides a mathematically and conceptually transparent framework that allows us to systematically analyse resonance-dependent interactions between spatial modes and their consequences for pattern stability and dynamics.

### 4.2. Velocity-Driven Asymmetry as Modulatory Input in Attractor Dynamics

As discussed in Section 3.2.2, the drift velocity of activity patterns is determined by the asymmetric component of the connectivity kernel under a fixed a condition. In the present model, the parameter a should be interpreted as an effective representation of velocity-driven modulation rather than a literal change in synaptic connectivity.

From a biological perspective, several experimental observations support the interpretation that motion-related signals act primarily as modulatory inputs to navigation circuits. For example, speed cells have been identified in the medial entorhinal cortex whose firing rates correlate approximately linearly with the animal’s running speed [34]. These neurons form a population distinct from grid cells and are believed to provide velocity-related inputs to the grid-cell network. Such signals are therefore likely to bias the dynamics of the network rather than fundamentally reshape its recurrent connectivity. Similarly, studies of the head-direction system have shown that vestibular inputs conveying angular velocity are essential for maintaining directional tuning [35,36]. These signals are generally interpreted as driving translations of an activity bump within a continuous attractor network rather than modifying the underlying connectivity structure.

Taken together, these experimental findings suggest that velocity-related signals in navigation circuits may act as relatively weak modulatory inputs that translate activity patterns within an existing attractor structure. In this interpretation, the asymmetry parameter a in our model represents a phenomenological mechanism through which velocity-dependent inputs bias the effective interactions in the neural field and induce gradual translations of the activity pattern. In the present analysis we assume that the asymmetry remains small, which allows the travelling-pattern dynamics to be studied within a perturbative framework while preserving the stability of the underlying spatial pattern. Biologically, this assumption is also consistent with the view that velocity signals act as modulatory inputs superimposed on a stable attractor structure rather than dominating the recurrent network dynamics.

### 4.3. From Pattern Dynamics to Spatial Representation

While the present study focuses on the dynamical mechanisms underlying pattern formation in neural fields, it is important to clarify how such patterns may relate to navigational computations. In attractor-based models of spatial representation, variables such as head direction or position are typically encoded by the phase of a population activity pattern. In this interpretation, the location of the activity bump (or the phase of a lattice pattern) represents the internal estimate of the corresponding spatial variable, and translational motion of the activity pattern corresponds to an update of the represented variable. For example, velocity inputs can induce shifts of the activity pattern across the neural manifold, thereby updating the encoded position through path integration. Similar principles have been proposed in attractor models of the head-direction system [7] and grid-cell networks [11].

From this perspective, the pattern dynamics analyzed in the present work provide a theoretical description of the mechanisms that can generate and stabilise such structured activity states. Rather than directly implementing navigational computations, the neural field dynamics studied here can be interpreted as defining the dynamical substrate upon which spatial representations and path-integration-like computations may operate. Importantly, this interpretation does not depend on a specific choice of connectivity kernel or activation function, but instead reflects general dynamical properties of continuous attractor systems near pattern-forming instabilities.

### 4.4. Biomimetic Applications

The neural field dynamics and pattern formation mechanisms analyzed in this study have direct implications for biologically inspired robotic systems, particularly in the domain of spatial representation and navigation. Biological grid cells and related spatial coding systems are widely believed to contribute to internal spatial representations that support navigation and path integration in mammals. In our previous work, neural representations inspired by grid cell dynamics were successfully implemented in virtual robotic navigation to yield robust localization and exploration behaviors [37], while the specific model used in that study differs from the neural field formulation analysed here. The present framework extends these concepts by elucidating how intrinsic network properties, such as synaptic kernel shape and nonlinear activation functions, influence the stability and morphology of emergent activity patterns. Such dynamics can be used in the design of neural-inspired control architectures for autonomous robots. The present study does not aim to implement a complete robotic navigation algorithm. Instead, it provides a theoretical framework that identifies dynamical principles of pattern formation and translation, which may serve as a foundation for future neural-inspired navigation architectures.

Specifically, the identified relationships between synaptic kernel structure and emergent pattern morphology provide a principled means of tuning spatial representations in artificial neural field controllers. For instance, by adjusting inhibitory/excitatory balance in neural circuits, one can engineer distributed activity patterns that encode grid-like spatial maps with desired scales and orientations, potentially supporting path-integration-like computations in robotic agents.

The bifurcation analysis highlights parameter regimes that promote robust pattern stability against perturbations. In practical robotic systems operating in noisy, unpredictable environments, such stability can be leveraged to maintain coherent spatial codes even when sensory inputs are degraded or intermittent. This could reduce reliance on expensive, high-precision sensors by instead exploiting intrinsic network dynamics for error correction in localization tasks.

Together, these observations suggest possible directions for translating neural field principles into computational architectures for distributed spatial representation and control in biomimetic and neuromorphic robotic platforms. Experimental implementation of the present neural field model in robotic systems remains an interesting direction for future work.

## 5. Conclusions

In this paper, we have examined how the interaction between nonlinear activation functions and lateral connectivity shapes spatially structured activity patterns in continuous attractor network (neural fields). By distinguishing between non-resonant and resonant critical modes, we show that resonance conditions give rise to a quadratic three-mode coupling that qualitatively modifies the amplitude dynamics. We identify a key parameter arising from the coupling mechanism that provides a concrete route for the generation of hexagonal lattice patterns. Beyond static pattern formation, we demonstrate that introducing a weak asymmetry in the connectivity kernel leads to a drift of spatial patterns, with the drift velocity determined explicitly by the asymmetric component of the coupling. When asymmetry component provided by external inputs, the resulting translations of population activity can link to inputs directly. Such mechanisms naturally account for activity-bump tracking in head-direction representations and lattice translations in grid-cell systems. Taken together, our results highlight how kernel–activation interactions govern both the selection and movement of spatial activity patterns, offering a unified dynamical interpretation of continuous representations in neural navigation systems and providing a theoretical foundation for translating neural field mechanisms into biomimetic and neuromorphic architectures for navigation.

## Figures and Tables

**Table 1:** Simulation parameters of the model.

Signal	Value	Significance
$A_{k}$	0.891	inhibition strength of $\hat{\omega }\left(k\right)$
$\sigma_{k}$	1.8	inhibition spatial range of $\hat{\omega }\left(k\right)$
μ	1.005∼1.06	bifurcation parameter
a	(0.03∼0.18, 0)	asymmetric displacement
δ	−0.26∼0.28	input bias of *F*/firing threshold
*b*	8	gain of activation function *F*
*N*	96	spatial grid
dt	0.03	time step of integration
$m_{\lambda }$	6	number of waves in simulation area
*L*	$\frac{2\pi m_{\lambda }}{k_{c}}$	size of simulation area
nsteps	≥4000	iteration steps

## Data Availability

The data supporting the findings of this study are available from the corresponding author upon reasonable request. Code will be made available on request.

## Appendix A

Although the local slope $s:=F^{\prime }\left(u_{0}\right)$ is determined by the fixed point, we treat the global gain μ as the bifurcation parameter and expand it about its critical value $\mu_{c}$ in the weakly nonlinear analysis. At second order we obtain

(A1) $$\begin{array}{c} O\left(\varepsilon^{2}\right):\left(I-\mu_{c}sW*\right)u_{2}=\frac{1}{2}f_{2}\mu_{c}W*\left(u_{1}^{2}\right). \end{array}$$

Notably, $\partial_{t}u_{2}=0$ in fast time scale. In Fourier space, the convolution operator W∗ acts as multiplication by $\hat{\omega }\left(|q|\right)$. For any non-critical wave vector q satisfies

(A2) $$\begin{array}{c} {\hat{u}}_{2}\left(q\right)=\frac{\frac{1}{2}f_{2}\mu_{c}\hat{\omega }\left(|q|\right)}{1-\mu_{c}s\hat{\omega }\left(|q|\right)}{\left({\hat{u}}_{1}^{2}\right)}_{q}, \end{array}$$

where $u_{1}^{2}=\sum_{i,j}\left(C_{i}C_{j}e^{i(k_{i}+k_{j})\cdot x}+C_{i}C_{j}^{*}e^{i(k_{i}-k_{j})\cdot x}+c.c.\right)$. Equation (A2) contains zero-frequency q=0, second-harmonic $q=2k_{n}$, synthetic-frequency $q=k_{i}+k_{j}\;\left(i\neq j\right)$, difference-frequency $q=k_{i}-k_{j},\;i\neq j$. Then we obtain:

(A3) $$\begin{array}{c} {\left({\hat{u}}_{1}^{2}\right)}_{0}=2\sum_{j}{\left|C_{j}\right|}^{2},\\ {\left({\hat{u}}_{1}^{2}\right)}_{2k_{n}}=C_{n}^{2},\\ {\left({\hat{u}}_{1}^{2}\right)}_{k_{i}+k_{j}}=C_{i}C_{j},\\ {\left({\hat{u}}_{1}^{2}\right)}_{k_{i}-k_{j}}=C_{i}C_{j}^{*}. \end{array}$$

The expression for each harmonic of $u_{2}$ can be solved by Equation (A2) when $1-\mu_{c}s\hat{\omega }\left(|q|\right)\neq 0$, i.e., q is a non-critical wave vector. For the hexagonal triad $\left\{k_{1},k_{2},k_{3}\right\}$ with $|k_{n}|=k_{c}$ and $k_{2}+k_{3}+k_{1}=0$, quadratic interactions generate critical-wavevector components (triad resonance), e.g., $k_{2}+k_{3}=-k_{1}$. In this situation, the expression for $u_{2}$ cannot be solved directly.

We collect $\varepsilon^{3}$-items:

(A4) $$\begin{array}{c} O\left(\varepsilon^{3}\right):\left(I-\mu_{c}sW*\right)u_{3}+\partial_{T}u_{1}=\mu_{2}sW*\left(u_{1}\right)+\mu_{c}\left(f_{2}W*\left(u_{1}u_{2}\right)+\frac{1}{6}f_{3}W*\left(u_{1}^{3}\right)\right). \end{array}$$

According to Fredholm’s condition,

(A5) $$\begin{array}{c} \partial_{T}C_{n}=\langle \mu_{2}sW*\left(u_{1}\right)+\mu_{c}f_{2}W*\left(u_{1}u_{2}\right)+\mu_{c}\frac{1}{6}f_{3}W*\left(u_{1}^{3}\right),e^{-ik_{n}\cdot x}\rangle . \end{array}$$

Since $\langle W*\left(u_{1}\right),e^{-ik_{n}\cdot x}\rangle =\langle u_{1},W*e^{-ik_{n}\cdot x}\rangle =\hat{\omega }\left(\right|k_{n}\left|\right)\langle u_{1},e^{-ik_{n}\cdot x}\rangle =\hat{\omega }\left(k_{c}\right)C_{n}$, and we use $|k_{n}|=k_{c}$, hence $\hat{\omega }\left(\right|k_{n}\left|\right)=\hat{\omega }\left(k_{c}\right)$. Another two items

(A6) $$\begin{array}{c} \langle W*\left(u_{1}^{3}\right),e^{-ik_{n}\cdot x}\rangle =\hat{\omega }\left(k_{c}\right)(3C_{n}|C_{n}{|}^{2}+6C_{n}\sum_{m\neq n}|C_{m}{|}^{2}),\\ \langle W*\left(u_{1}u_{2}\right),e^{-ik_{n}\cdot x}\rangle =\hat{\omega }\left(k_{c}\right)\;coeff\left(u_{1}u_{2}\right), \end{array}$$

where

(A7) $$\begin{array}{c} coeff\left(u_{1}u_{2}\right)=C_{n}(2A_{0}\sum_{j}|C_{j}{|}^{2}+A_{2}|C_{n}{|}^{2}+\sum_{m\neq n}\left(2A_{-}^{\left(m\right)}+2A_{+}^{\left(m\right)}\right)|C_{m}{|}^{2}),\\ A_{0}=\frac{\mu_{c}f_{2}\hat{\omega }\left(0\right)}{1-\mu_{c}s\hat{\omega }\left(0\right)},\;A_{2}=\frac{\mu_{c}f_{2}\hat{\omega }\left(2k_{c}\right)}{1-\mu_{c}s\hat{\omega }\left(2k_{c}\right)},\\ A_{-}^{\left(m\right)}=\frac{\mu_{c}f_{2}\hat{\omega }\left(\right|k_{n}-k_{m}\left|\right)}{1-\mu_{c}s\hat{\omega }\left(\right|k_{n}-k_{m}\left|\right)},\;A_{+}^{\left(m\right)}=\frac{\mu_{c}f_{2}\hat{\omega }\left(\right|k_{n}+k_{m}\left|\right)}{1-\mu_{c}s\hat{\omega }\left(\right|k_{n}+k_{m}\left|\right)}. \end{array}$$

Combine and extract auto-coupling $C_{n}{\left|C_{n}\right|}^{2}$ and mutual-coupling $C_{n}{\left|C_{m}\right|}^{2}$ terms, we obtain

(A8) $$\begin{array}{c} \partial_{T}C_{n}=C_{n}(\gamma +(c_{11}|C_{n}{|}^{2}+c_{12}|C_{m}{|}^{2})),\\ \gamma =\mu_{2}s\hat{\omega }\left(k_{c}\right),\\ c_{11}=\mu_{c}\hat{\omega }\left(k_{c}\right)\;\left(\frac{1}{2}f_{3}+f_{2}\left(2A_{0}+A_{2}\right)\right),\\ c_{12}=\mu_{c}\hat{\omega }\left(k_{c}\right)\;\left(f_{3}+f_{2}\left(2A_{0}+2A_{-}^{\left(m\right)}+A_{+}^{\left(m\right)}\right)\right). \end{array}$$

To unify the form, we rewritten

(A9) $$\begin{array}{c} \partial_{T}C_{n}=C_{n}(\gamma -\alpha |C_{n}{|}^{2}-2\beta \left|C_{m}{|}^{2}\right), \end{array}$$

where $\alpha =-c_{11},\beta =-\frac{c_{12}}{2}$.

## Appendix B

For hexagonal patterns, three critical wavevectors $\left\{k_{1},k_{2},k_{3}\right\}$ satisfy $k_{1}+k_{2}+k_{3}=0$. In this case, quadratic nonlinearities generate resonant interactions that project directly onto the critical subspace. To capture this effect, we introduce a faster slow time $T_{1}=\varepsilon t$, $\partial_{t}\mapsto \partial_{t}+\varepsilon \partial_{T_{1}}+\varepsilon^{2}\partial_{T_{2}}$, and $\mu =\mu_{c}+\varepsilon \mu_{1}+\varepsilon^{2}\mu_{2}$.

At O(ε) we obtain

(A10) $$L_{\mu_{c}}u_{1}=0,\;L_{\mu_{c}}=-I+\mu_{c}s\;W*,$$

so that $u_{1}$ lies in the critical eigenspace. Hence

(A11) $$u_{1}\left(x,T_{1},T_{2}\right)=\sum_{n=1}^{3}\left(C_{n}\left(T_{1},T_{2}\right)e^{ik_{n}\cdot x}+C_{n}^{*}\left(T_{1},T_{2}\right)e^{-ik_{n}\cdot x}\right).$$

At $O(\varepsilon^{2})$ the governing equation reads

(A12) $$\partial_{T_{1}}u_{1}=L_{\mu_{c}}u_{2}+\mu_{1}s\;W*u_{1}+\mu_{c}\frac{f_{2}}{2}\;W*\left(u_{1}^{2}\right).$$

Using the resonance condition $k_{n-1}+k_{n+1}=-k_{n}$, the quadratic term $u_{1}^{2}$ contains a contribution proportional to $C_{n-1}^{*}C_{n+1}^{*}e^{ik_{n}\cdot x}$. After convolution, this gives

(A13) $$\mu_{c}\frac{f_{2}}{2}W*\left(u_{1}^{2}\right)\;⟶\;\mu_{c}f_{2}\;\hat{\omega }\left(k_{c}\right)\;C_{n-1}^{*}C_{n+1}^{*}e^{ik_{n}\cdot x}.$$

Therefore, $\eta =\mu_{c}f_{2}\;\hat{\omega }\left(k_{c}\right)$.
